# Geospatial Distribution of Tuberculosis Incidence and Determinants of Tuberculosis Treatment Outcomes in Nzema East Municipality, Ghana

**DOI:** 10.1002/puh2.70108

**Published:** 2025-08-12

**Authors:** Charles Afriyie Agyapong, Ali Davod Parsa, Richard Hayhoe, Russell Kabir, Mark Cortnage

**Affiliations:** ^1^ School of Allied Health Anglia Ruskin University Chelmsford Essex UK; ^2^ Ghana Health Service Accra Ghana

**Keywords:** geospatial pattern, Ghana, Nzema East, treatment outcome, tuberculosis

## Abstract

**Background:**

Ghana has seen a notable rise in tuberculosis (TB) cases with mired treatment outcomes. However, evidence suggests disparities in the incidence of TB and its treatment outcomes across the country. Nzema East Municipality specifically reported a 62.34% increase in TB incidence in 2023 compared to 2022. The study, therefore, aims to determine the geospatial distribution of TB incidence and predictors of TB treatment outcomes in Nzema East Municipality.

**Methods:**

The study used a retrospective cohort with a quantitative approach, utilising health records of 545 TB cases from 2018 to 2023 in Nzema East. Data were processed with Microsoft Excel and analysed using ArcGIS Pro version 3.3.2, Joinpoint Regression Programme 5.2.0 and STATA MP version 17.

**Results:**

The Moran's index was 0.03 (*p* < 0.001). All the subdistricts had at least one settlement with 2–26 TB cases per square kilometre. Significant TB hotspots were identified in the population‐dense communities and mining communities. Overall, the successful TB treatment outcome was 76.70%. There was a significant decline in successful TB treatment outcomes from 2018 to the end of 2020 and through 2023 (*p* = 0.03 and *p* < 0.001), respectively. Having at least one follow‐up lab (aRR = 0.43; 95% CI = 0.32, 0.58) and having a treatment supporter (aRR = 0.56; 95% CI = 0.40, 0.79) lessens the risk of having an unsuccessful TB treatment outcome. Having started the TB treatment in 2020 increases the chances of having an unsuccessful outcome (aRR = 1.97; 95% CI = 1.13, 3.43).

**Conclusion:**

TB incidence in Nzema East was spatially dependent, with statistically significant higher incidence in the highly populated and mining communities. The overall successful treatment outcome is suboptimal, which demands targeted intervention to mitigate these menaces.

## Graphical abstract

This study revealed significant spatial clustering of tuberculosis cases in Ghana's Nzema East Municipality, with hotspots in mining and densely populated communities. Treatment success rate (76.7%) fell below WHO targets. Follow‐up laboratory testing and treatment supporters improved outcomes, whereas COVID‐19 period treatments showed increased failure rates, demanding targeted interventions.

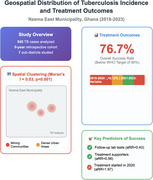

## 1 Introduction

### 1.1 Background

Tuberculosis (TB), a severe and highly contagious disease, presents a significant global health challenge, being the leading cause of mortality from a single infectious disease among humans globally. The need for collective action to curb this challenge is urgent, given its global burden of approximately 1.3 million mortalities in 2023, surpassing deaths attributed to COVID‐19 and about twice the deaths caused by HIV and AIDS [1]. In 2023, over 10.8 million individuals worldwide were diagnosed with active TB, of which 8.2 million were newly diagnosed, unacceptably higher than the 7.5 million in 2022 [1].

TB is caused by the bacillus *Mycobacterium tuberculosis* (MTB) and can affect any part of the body, although it primarily affects the lungs (known as pulmonary TB). It can also affect individuals of all age groups. The MTB from an actively infected lung TB person can get to those without the bacillus through the air when they cough, sneeze or spit [2]. Individuals who have been infected with MTB might not exhibit symptoms of TB, a condition commonly referred to as latent TB. Approximately 25% of the world's population have latent TB, giving them a concerning chance of developing active TB [3, 4]. About 10% of persons with latent TB acquire active TB [5]. Moreover, those with compromised immune systems are at a greater risk of developing active TB disease [6]. Approximately 6.1% of the global active TB cases were people living with HIV [1].

Efforts in controlling this deliberating disease led to the adoption of the End TB Strategy of the World Health Organisation (WHO) through integrated, patient‐centred care and prevention, bold policies and supportive systems and intensified research and innovations; and the United Nations Sustainable Development Goals 2015, to end this TB epidemic [1]. The WHO's End TB Strategy recommends reducing 90% of TB incidence and 95% of TB deaths by 2035 compared to 2015 by all nationals or other implementing bodies, with one of the monitoring targets for implementation of this strategy being at least 90% in TB treatment success rate [1, 7].

Unfortunately, in Ghana, TB remains a consistent burden, causing substantial health and economic implications for both individuals and the overall healthcare system, despite ongoing efforts to mitigate its impact. As indicated in the 2022 Ghana Holistic Assessment Report, there has been a notable increase in TB cases in Ghana. Specifically, between 2018 and 2022, the incidence of new TB infections has risen by 13.2%, that is, 14,602–16,526, with the number of new cases per 100,000 people increasing from 43.4% to 52.5% [8].

Nevertheless, in addition to curbing the rising TB incidence, there is a higher demand for increasing the successful treatment of persons with TB in Ghana, as the treatment outcomes stagnated at 87% from 2018 to 2022 [8]. This poor TB treatment outcome could be predicted by clinicodemographic characteristics such as age, sex and HIV infection [9, 10]. It is worth noting that successful treatment of TB is not only necessary for just its treatment or elimination but also to prevent other public health issues, such as TB‐associated morbidity and antimicrobial resistance [11, 12, 13].

TB data are frequently presented as a single national incidence or prevalence. It is imperative to analyse the TB data at a primary level to gain a more comprehensive understanding of the disease's burden in a particular region or community, which may differ from that of the larger national population.

Nzema East Municipality, one of the 14 districts of the Western Region, Ghana, is highly burdened with TB. Of the about 2000 new TB cases reported (both new and relapse) in the Western Region in 2023, 154 (i.e., 156 cases per 100,000 population) were from Nzema East, a 62.34% increase in new cases from what was recorded in the municipality in 2022 [14]. This rise in the incidence of TB cases in the municipality is unacceptable, as it directly impedes Nzema East from contributing its quota to achieving the 90% reduction in TB incidence recommended by the WHO [1]. As such, tailored interventions are urgently needed to prevent and control TB in the municipality.

Despite this pressing issue, there remains a critical gap in understanding the geospatial dynamics of TB incidence and factors affecting treatment outcomes within the Nzema East. This gap hinders the development and implementation of effective, evidence‐based interventions to mitigate the burdens of TB in the municipality. Hence, this study seeks to address this gap by determining the geospatial distribution of TB incidence and identifying predictors of TB treatment outcomes in Nzema East Municipality.

## 2 Methodology

### 2.1 Study Site Description

This study was conducted in the Nzema East Municipality in the Western Region of the Republic of Ghana. It is located between longitude 2° 05′ and 2° 35′ west and latitude 4° 40′ and 5° 20′ north, covering a land area of 2194 km^2^. Nzema East is bounded by Ellembelle District to the west, hanta West to the east, Tarkwa Nsuem Municipal to the north and the Gulf of Guinea to the south.

The municipality's inhabitants in the northern part are predominantly farmers and small‐scale miners (including Galamsey), whereas those who reside in the southern part are fishers and fishmongers. The district's population is projected to be 98,851 in 2023, based on the 2010 Ghana Population and Housing Census. The population density is higher in the southern sector of the municipality, which is, incidentally, more endowed with social amenities such as safe and reliable drinking water sources, schools, health facilities and electricity.

For health administrative purposes, the municipality is divided into 7 subdistricts, with 1 municipal hospital, 4 health centres and 12 Community‐Based Health Planning and Services (CHPS) zones as of the end of 2023. The municipality has only one certified diagnostic centre for TB, the Municipal Hospital located in Axim, the municipality's capital.

### 2.2 Study Design and Approach

This study adopted a retrospective cohort study of the new TB cases in Nzema East from 1 January 2018 to 31 December 2023, with a quantitative approach. The retrospective cohort study design allowed the use of secondary data for geospatial analysis and to identify the factors predicting the treatment outcomes of TB cases [15, 16].

### 2.3 Study Population

The study population included all newly diagnosed drug‐susceptible TB cases treated in Nzema East Municipality from 1 January 2018 to 31 December 2023. Out of the 559 total cases registered during this period, 545 met the inclusion criteria and were enrolled, achieving a 97.5% coverage rate of the target population.

#### 2.3.1 Inclusion Criteria

A case was only included in a study when diagnosed and initiated their treatment from 1 January 2018 to 31 December 2023.

#### 2.3.2 Exclusion Criteria

A case was exempted from the study if they had no health records in the Nzema East TB register, were referred to continue their treatment in any facility outside Nzema East Municipality or were still undergoing treatment at the time of the study.

#### 2.3.3 TB Case Definitions and Diagnostic Methods

Cases in this study were diagnosed through either bacteriological confirmation or clinical assessment following WHO guidelines.

Bacteriologically confirmed cases were diagnosed through:

1. sputum smear microscopy (Ziehl‐Neelsen staining) for acid‐fast bacilli;
2. GeneXpert MTB/RIF assay for MTB detection and rifampicin resistance screening.

Clinically diagnosed cases were identified on the basis of:

1. typical TB symptoms (persistent cough >2 weeks, fever, night sweats, weight loss);
2. chest x‐ray findings consistent with TB;
3. clinical improvement following anti‐TB treatment initiation.

Drug susceptibility was presumed for new cases with:

1. no previous TB treatment history;
2. no rifampicin resistance on GeneXpert testing where available;
3. no clinical evidence suggesting drug resistance.

### 2.4 Sampling

**FIGURE 1:**
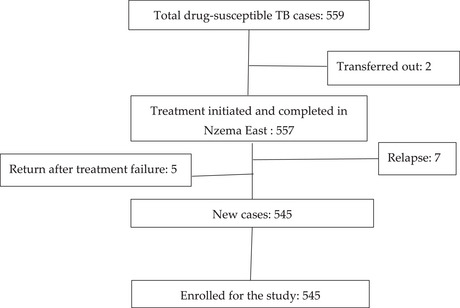
Flow chart of sampling of the study participants. TB, tuberculosis.

**FIGURE 2:**
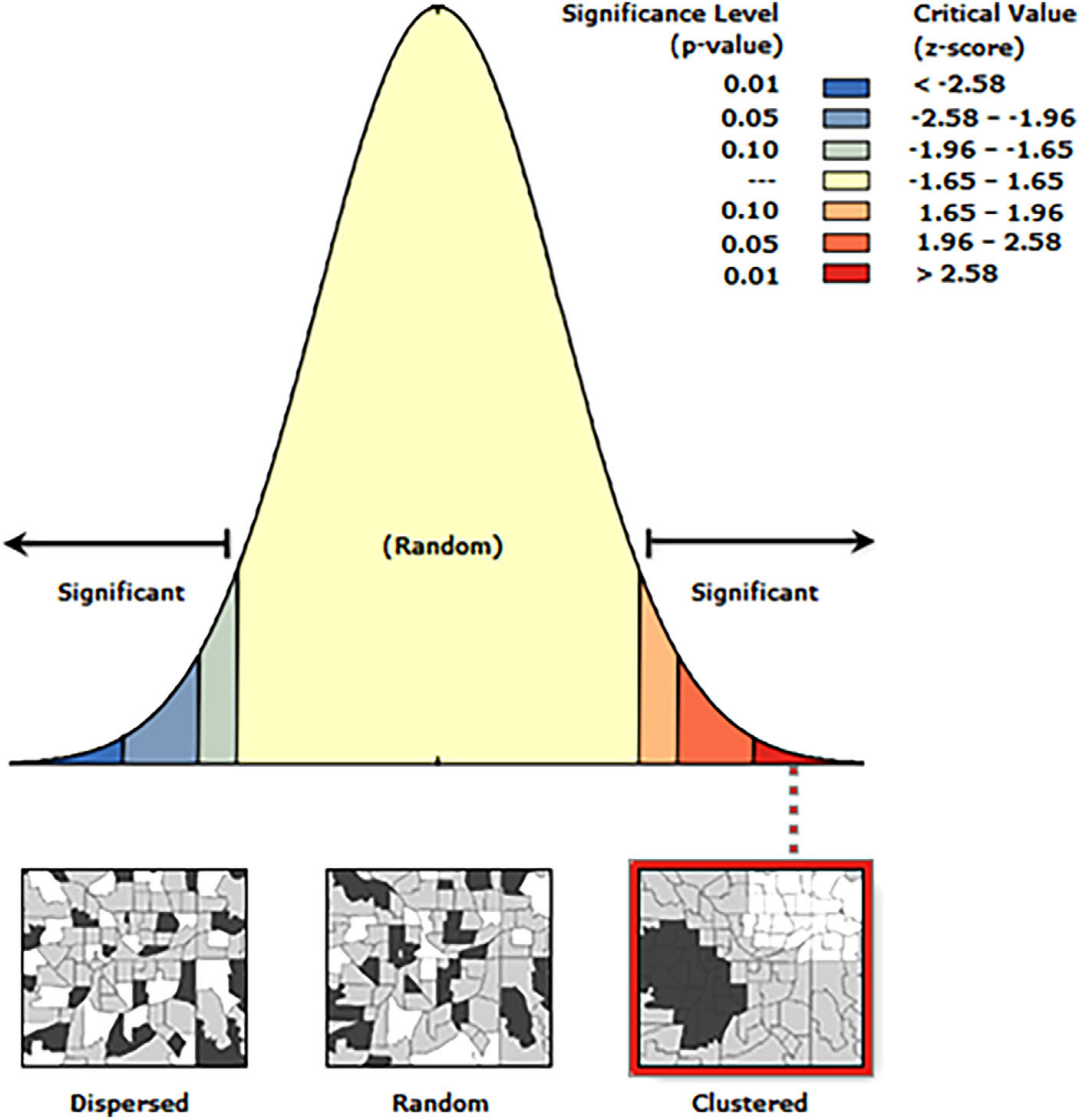
Correlation of the TB cases in Nzema East Municipality.

Using a total population sampling technique for this study, 545 cases were eligible and were enrolled for this study (Figure 1 and 2).

### 2.5 Study Variables

The dependent variables are TB incidence distribution and TB treatment outcome. The treatment outcome variable is grouped into successful treatment and unsuccessful treatment outcomes. Cases who were declared cured or complete as at the time of the study were identified as having a successful treatment outcome. Unsuccessful treatment outcomes included cases who were declared lost to follow‐up, died or had treatment failure and those who were not evaluated as of the time of the study. The independent variables are age, gender, date of TB treatment, disease site, follow‐up test results, HIV status, subdistrict of residents, town or community of residents, type of location of residents (either urban or rural) and XY coordinates of the place of residents.

### 2.6 Data Collection Techniques and Tools

This study used secondary data from the Nzema East TB case registers for January 2018 to December 2023. The data were collected retrospectively at a single time point in January 2024, after the study period ended.

Eight trained research assistants, who were healthcare professionals in Nzema East, extracted the required variables from the TB registers into a standardised Microsoft Excel template over a 2‐week period. Regular meetings ensured consistency in the data extraction process.

The extracted data were then validated by the Nzema East Municipal TB Programme Coordinator to verify accuracy and completeness, with any discrepancies resolved through discussion and cross‐referencing with the original registers. The final validated dataset was used for analysis.

### 2.7 Data Analysis

The data were analysed on the basis of the specific objectives and depicted in tables and figures as appropriate.

#### 2.7.1 Analysing the Spatial Distribution of the TB Cases

The extracted data were exported into Esri ArcGIS Pro version 3.3.2 for analysis. The cases were georeferenced on the basis of their residence address recorded in the register, which was used to identify their statistically significant distribution. All feature classes were projected into the Ghana Metre Grid coordinate system with metres as a unit of measurement. Spatial autocorrelation (Global Moran's I) was used to assess the spatial dependence of the TB cases, and the values were reported as *z*‐scores, *p* values and Moran's index.

The global Moran's I spatial autocorrelation was computed as [17]

$$M=\frac{N}{W}\times \frac{W\;\left(x_{y}-\bar{x}\right)\left(x_{z}-\bar{x}\right)}{\sum_{y=1}^{N}{\left(x_{y}-\bar{x}\right)}^{2}}$$

$$W=\sum_{y=1}^{N}\sum_{z=1}^{N}S_{yz}$$

where *M* is the global Moran's index, *N* is the total number of spatial units, *x_y_* and *x_z_* are the number of TB incidences in settlements *y* and *z*, respectively, *W* is the sum of spatial weights, $\bar{x}$ is the average number of TB incidence and *S_yz_* is the spatial weight between settlements *y* and *z*. The global Moran's I statstic quantifies spatial autocorrelation by assessing the randomness of the spatial pattern, with the index taking values from −1 to +1. The positive values denote spatial clustering; negative values suggest spatial dispersion and the values near zero signify a random spatial pattern [18]. The statistical significance observed in the Moran's I was estimated using a *z*‐score, expressed mathematically as [18]

$$Z_{M}=\frac{M-E\;\left(M\right)}{\sqrt{V\;\left(M\right)}}$$

where $Z_{M}$ is the observed *z*‐score, E(M) is the expected value under the null hypothesis of spatial randomness and V(M) is the variance of Moran's I (*M*).

Kernel density estimation was performed to determine the magnitude of the occurrence of TB cases per square kilometre across the various settlements in the municipality. The data were further analysed using the Hotspot Analysis (Getis‐Ord Gi*) tool to identify the statistically significant hotspot zones for the TB incidence in Nzema East Municipality. Statistical significance was estimated as a *p* value less than 0.05 at a 95% confidence interval.

#### 2.7.2 Analysing the Trend of TB Treatment Outcomes Over Time

The data were exported to the US National Cancer Institute Joinpoint Regression Programme software version 5.2.0 to identify significant changes in TB treatment outcomes in Nzema East Municipality over time, which uses the Monte Carlo permutation method [19]. The data were aggregated into 6‐month intervals for the 6‐year study period, analysed and reported as half‐year percentage change (HPC), *p* values and confidence intervals, defining significance as *p* value less than 0.05 at a 95% confidence interval.

#### 2.7.3 Analysing for Predictors of Treatment Outcomes

Pearson chi‐square or Fisher's exact test was used to determine the independence of the variables on the TB treatment outcomes, with significance set at *p* value less than 0.05 at a 95% confidence interval. The Fisher's exact test was used for variables with at least a cell frequency less than 5 [20]. Modified Poisson regression with a robust standard error was further done to estimate the relative risk (RR) of unsuccessful TB treatment outcome with the independent variables and adjusted for potential confounding, which was computed as [21]

$$\log(E\;\left[Y_{i}|X_{i}]\right)=\beta_{0}+\beta_{1}X_{i1}+\beta_{2}X_{i2}+\beta_{3}X_{i3}+\cdots +\beta_{k}X_{ik}$$

where $Y_{i}$ is the treatment outcome (which was binary), $X_{i}$ ($X_{i1},\;X_{i2},\;X_{i3},\cdots ,\;X_{ik}$) are the predictor variables, *β* $(\beta_{0},\;\beta_{1},\;\beta_{2,}\;\beta_{3,}\cdots ,\;\beta_{k}$) are the regression coefficients and $E[Y_{i}X_{i}]$ is the expected probability. Because the model uses a log link function, the exponential coefficients ($\exp(\beta_{i}))$ directly estimates the RR with each predictor ($X_{i}$), expressed as [21]

$$RR_{i}=\exp\left(\beta_{i}\right)$$

Following the model estimation, robust (sandwich) standard error was employed to assume a potential misspecification of variance structure [22]. The robust variance–covariance matrix of the estimated coefficients $\hat{\beta }$ was expressed as [21]

$${\left(\hat{Var}\right)}_{r}\left(\hat{\beta }\right)=F^{\left(-1\right)}JF^{\left(-1\right)}$$

where *F* is the Fisher information matrix and *J* is the outer product of residuals. The confidence intervals for $RR_{i}$ are specified as

$$\exp(\hat{\beta_{i}}\pm 1.96\times {\sqrt{\hat{Var_{r}}\beta }}_{i})$$

Modified Poisson regression is appropriate for this study because the outcome is binary, and it allows direct estimation of RR for a common outcome, whereas odds ratios from logistic regression tend to overestimate RR [23]. Although log‐binomial regression is an alternative model that can directly estimate RR, modified Poisson regression with a robust standard error provides greater computational stability. It is not subject to convergence issues and allows for statistically valid inference while accounting for overdispersion and misspecification of the variance [21, 22, 24, 25].

The variables for adjustment in the modified Poisson regression were selected on the basis of statistical and subject‐matter considerations. A preliminary screening was conducted utilising a *p* value threshold of <0.20 in the Pearson chi‐square or Fisher's exact test [26]. The final model incorporated factors believed to be confounders, informed by prior literature, irrespective of the statistical significance in the bivariate analysis. The HIV status of the cases was intentionally incorporated into the final model despite failing to satisfy the *p* < 0.20 because of its probable role as a confounder in previous studies [27, 28, 29, 30].

### 2.8 Ethical Considerations

The study obtained ethical approval from the Committee on Human Research, Publications and Ethics, Kwame Nkrumah University of Science and Technology, Ghana (CHRPE/AP/336/24), and from the Anglia Ruskin University Research Ethics Board, UK (ETH2324‐6200). Moreover, local permission and approval for the study were sought from the Nzema East Municipal Health Administration using a formal letter of introduction.

## 3 Results

### 3.1 Demographic Characteristics

A total of 545 TB cases were followed for this study. Among these 545, an estimated 68.26% were males. The youngest case was 4 years old, and the average age among the cases was 41. About 60.00% of the cases were from the rural communities (Table 1).

**TABLE 1:** Demographic characteristics of the cohort.

Characteristics	Frequency (*n* = 545)	Percentage (%)
**Age group (years)**:		
4–14	8	1.47
15–24	60	11.01
25–34	116	21.28
35–44	150	27.52
45–54	130	23.85
55+	81	14.86
Mean age (SD):	41 (14.16)	
**Gender**:		
Female	173	31.74
Male	372	68.26
**Residence category**:		
Rural	327	60.00
Urban	218	40.00

### 3.2 Medical Information of the Cohort

Almost all (97.43%) of the cohort were diagnosed as pulmonary. About 427 (78.35%) of the 545 cases had at least one follow‐up lab during treatment. The majority (28.25%) of the cohort started treatment in 2023. Approximately 8.81% of the cases were HIV‐positive (Table 2).

**TABLE 2:** Clinical characteristics of the study group.

Characteristics	Frequency (*n* = 545)	Percentage (%)
**Infection site**:		
Extra pulmonary	14	2.57
Pulmonary	531	97.43
**At least one follow‐up lab**:		
No	118	21.65
Yes	427	78.35
**Year of treatment start**:		
2018	68	12.48
2019	85	15.6
2020	63	11.56
2021	79	14.5
2022	96	17.61
2023	154	28.25
Treatment support: No Yes	57 488	40.46 89.54
**HIV status**:		
Negative	497	91.19
Positive	48	8.81

### 3.3 Spatial Distribution of the TB Cases

#### 3.3.1 Global Moran's I Summary

The TB incidence in Nzema East Municipality has a clustered pattern. The clustered spatial inference of the TB cases was statistically significant (*p* < 0.001) (Table 3).

**TABLE 3:** Spatial correlation summary.

Characteristics	Value
Moran's index	0.03
*z*‐score	19.04
*p* value	0.00

#### 3.3.2 Case Density and Hotspots

All the sub‐municipalities of Nzema East had at least one settlement highly dense with TB cases (2–26 TB‐infected persons within a km^2^ range). Axim–Nsein and Dadwen–Kegyina sub‐municipalities had the majority of their settlements with 2–26 TB‐infected persons per km^2^. The Axim and Nsein settlements were inferred to be the major significant hotspot zones for TB incidence in the municipality. Other hotspot zones were found in Bamiankor, Apataim, Fantekrom and Avlebo.

**FIGURE 3:**
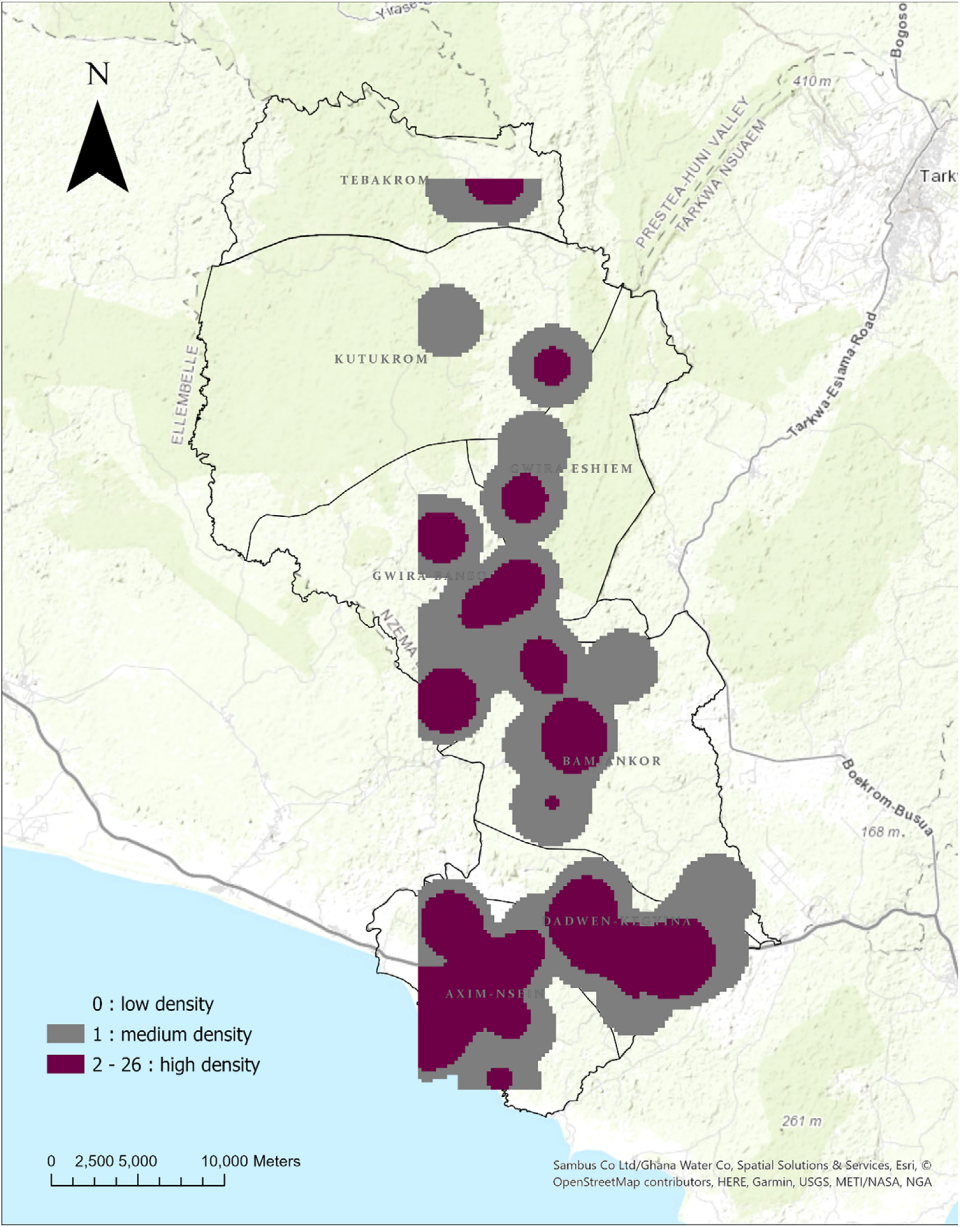
Density of TB cases in Nzema East, 2018–2023.

**FIGURE 4:**
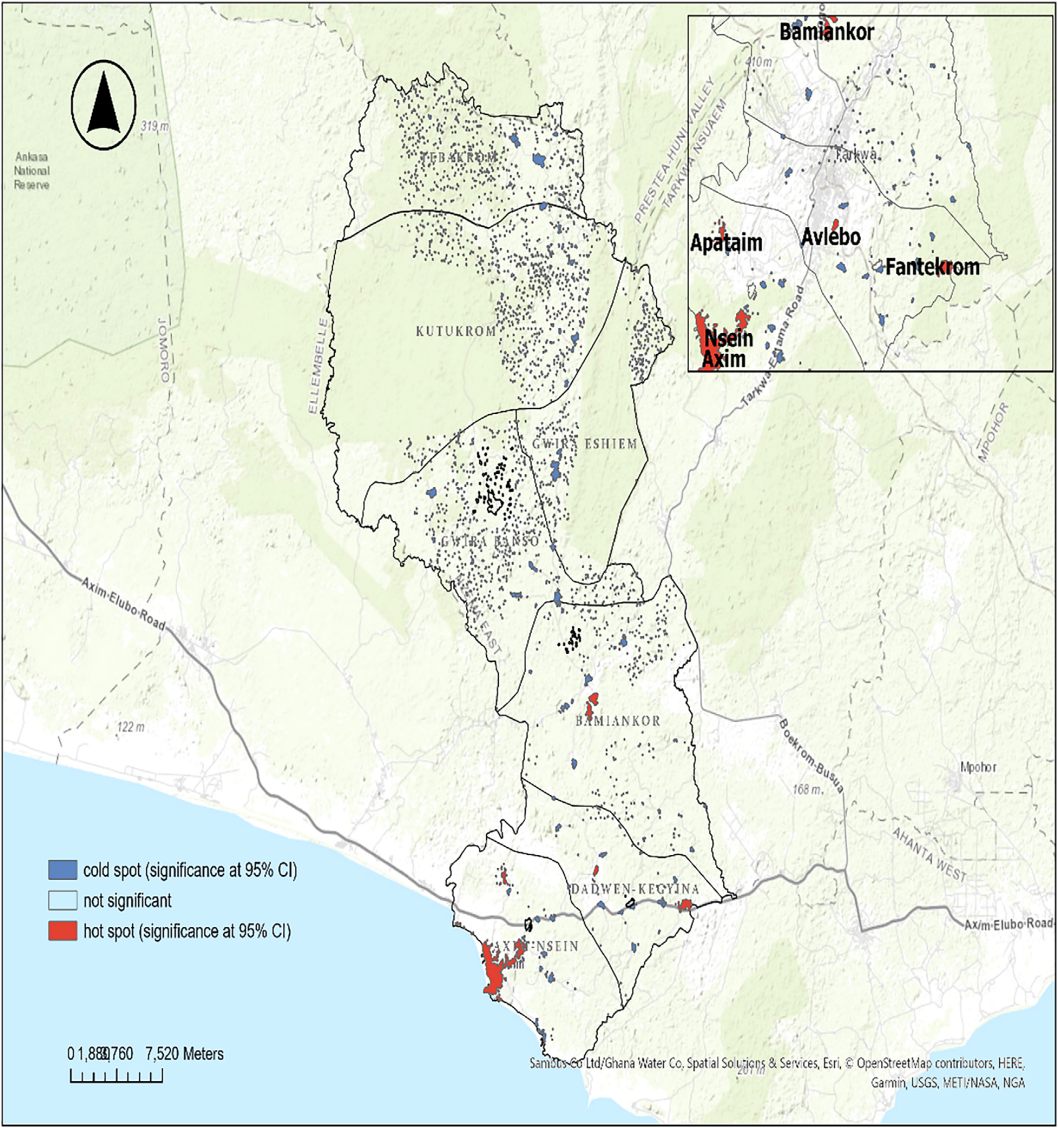
Hotspot zones of TB incidence in Nzema East.

Figure 3 shows the density of TB cases across the municipality. Figure 4 shows the statistically significant TB hotspot and cold spot zones in Nzema East Municipality.

### 3.4 TB Outcome Treatment Over Time

**FIGURE 5:**
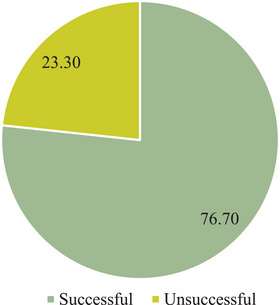
Overall TB treatment outcome.

Overall, from 2018 to 2023, Nzema East recorded a 76.70% TB treatment success rate (Figure 5).

**FIGURE 6:**
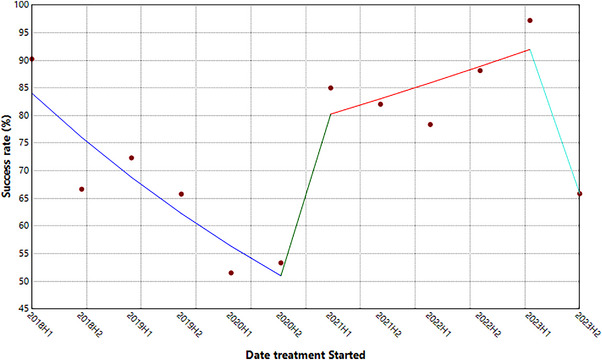
Trend in successful TB treatment outcomes.

The Joinpoint Regression model found three join points (Figure 6). The trend was segmented into four changes. Details of the segments are depicted in Table 4. There was a statistically significant 18.12% decrease in the successful treatment outcome from the first half‐year of 2018 to the end of 2020 (*p* = 0.03). From the second half‐year of 2020 to the first half‐year of 2021, the municipality observed a 147.88% upward successful TB treatment rate. The change was statistically insignificant (*p* = 0.14). A 7.04% increase in the successful TB treatment outcome was found from the first half‐year of 2021 to the first half‐year of 2023. The change was statistically insignificant (*p* = 0.14). There was a significant 48.74% decrease in successful treatment from 2023H1 to 2023H2 (*p* < 0.001).

**TABLE 4:** Trend in the tuberculosis (TB) treatment outcomes, 2018–2023.

Segment	HPC	95% CI	*p* value
2018H1–2020H2	−18.12^a^	−29.25, −9.03	0.03
2020H2–2021H1	147.88	−29.53, 236.13	0.14
2021H1–2023H1	7.04	−3.20, 140.58	0.14
2023H1–2023H2	−48.74^a^	−65.59, −6.79	<0.001

Abbreviation: HPC, half‐year percentage change.

^a^ The half‐year percentage change is statistically significant at 95% CI.

### 3.5 Predictors of Successful TB Treatment Outcome

#### 3.5.1 Cross‐Tabulation of Demographics and Treatment Outcome

There was no significant association between any of the demographic characteristics and the TB treatment outcomes. A comparative majority (24.41%) of the cohort who had unsuccessful treatment outcomes were from the ages of 25 to 34. The paediatrics (those aged below 15) contributed to 1.57% of those not successfully treated. More than half (64.57%) of the study group who were unsuccessfully treated for TB were males (Table 5).

**TABLE 5:** Contingency table of demographics and tuberculosis (TB) treatment outcomes.

Characteristics	Treatment outcome		*χ* ^2^	*p* value
	Successful	Unsuccessful		
**Age group**:			8.19	0.15^a^
<15	6 (1.44)	2 (1.57)		
15–24	49 (11.72)	11 (8.66)		
25–34	85 (20.33)	31 (24.41)		
35–44	122 (29.19)	28 (22.05)		
45–54	102 (24.40)	28 (22.05)		
55+	54 (12.92)	27 (21.26)		
**Gender**:			1.04	0.31
Female	128 (30.62)	45 (35.43)		
Male	290 (69.38)	82 (64.57)		
**Residents’ category**:				
Rural	243 (58.13)	84 (66.14)	2.60	0.11
Urban	174 (41.87)	43 (33.86)		

^a^ Fisher's exact test was used.

#### 3.5.2 Cross‐Tabulation of Medical Characteristics and Treatment Outcomes

Table 6 depicts the association between the cohort's medical characteristics and treatment outcomes. The association between having at least one follow‐up lab or not during treatment and the treatment outcomes was statistically significant (*χ* ^2^ = 36.34, *p* < 0.001). The year treatment started for the cohort was significantly associated with the TB treatment outcomes (*χ* ^2^ = 30.53, *p* < 0.001). A majority (91.34%) of the cohort who had an unsuccessful TB treatment were HIV‐negative. Having a treatment supporter was significantly associated with TB treatment outcome (*χ* ^2^ = 10.35, *p* < 0.001). An estimated 81.89% of the cohort who had treatment supporters were unsuccessfully treated for TB.

**TABLE 6:** Contingency table of the medical characteristics and tuberculosis (TB) treatment outcomes.

Characteristics	Treatment outcome		*χ* ^2^	*p* value
**Infection site**:	Successful	Unsuccessful		
Extra pulmonary	12 (2.87)	2 (1.57)	0.65	0.33^a^
Pulmonary	406 (97.13)	125 (98.43)		
**At least one follow‐up lab**:				
No	66 (15.79)	52 (40.94)	36.34	<0.001^*^
Yes	352 (84.21)	75 (59.06)		
**Year of treatment start**:				
2018	55 (13.16)	13 (10.24)	30.53	<0.001*
2019	59 (14.11)	26 (20.47)		
2020	33 (7.89)	30 (23.62)		
2021	66 (15.79)	13 (10.24)		
2022	81 (19.38)	15 (11.81)		
2023	124 (29.67)	30 (23.62)		
**Treatment support**:				
No	34 (8.13)	23 (18.11)	10.35	<0.001*
Yes	384 (91.87)	104 (81.89)		
**HIV status**:				
Negative	381 (91.15)	116 (91.34)	0.03	0.94
Positive	37 (8.85)	11 (8.66)		

^a^ Fisher's exact test was used.

* Statistically significant at 95% CI.

#### 3.5.3 Modified Poisson Regression Predicting the Factors Associated With Unsuccessful TB Treatment Outcome

The cohort aged 55 years and over had a 21% increased risk of having unsuccessful treatment compared to the paediatrics (aRR = 1.21; 95% CI = 0.42, 3.47). The participants who reside in the urban settlements had a 20% lower risk of having an unsuccessful treatment outcome compared to those in the rural settlements (aRR = 0.80; 95% CI = 0.58, 1.08). The cases who had at least one follow‐up lab during treatment had 57% less risk of having unsuccessful treatment compared to those who never had any follow‐up lab during treatment (aRR = 0.43; 95% CI = 0.32, 0.58). The cases who started their treatment in 2020 had a 97% increased risk of having unsuccessful treatment outcomes compared to those who started theirs in 2018 (aRR = 1.97; 95% CI = 1.13, 3.43). Cases who had treatment supporters had a 44% decreased risk of having unsuccessful treatment outcomes compared to those who did not (aRR = 0.56; 95% CI = 0.40, 0.79). The HIV co‐infected TB clients had a 15% lower risk of having an unsuccessful treatment outcome compared to the TB clients without HIV infection (aRR = 0.85; 95% CI = 0.53, 1.38).

Table 7 shows detailed predictors of unsuccessful TB treatment outcomes among the cohort.

**TABLE 7:** Associated factors of unsuccessful treatment outcome.

Characteristics	Treatment outcome (unsuccessful)	
	cRR [95% CI]	aRR [95% CI]
**Age group**:		
<15	1.00_(ref)_	1.00_(ref)_
15–24	0.73 [0.20, 2.73]	0.77 [0.25, 2.39]
25–34	1.07 [0.31, 3.69]	0.94 [0.33, 2.70]
35–44	0.75 [0.21, 2.60]	0.76 [0.26, 2.20]
45–54	0.86 [0.25, 2.99]	0.83 [0.29, 2.38]
55+	1.33 [0.39, 4.61]	1.21 [0.42, 3.47]
**Gender**:		
Female	1.00_(ref)_	
Male	0.85 [0.62, 1.16]	
**Residents’ category**		
Rural	1.00_(ref)_	1.00_(ref)_
Urban	0.77 [0.55, 1.06]	0.80 [0.58, 1.08]
**Infection site**:		
Extra pulmonary	1.00_(ref)_	
Pulmonary	1.65 [0.45, 6.01]	
**At least one follow‐up lab**:		
No	1.00_(ref)_	1.00_(ref)_
Yes	0.40 [0.30, 0.53]*	0.43 [0.32, 0.58]*
**Year of diagnosis**:		
2018	1.00_(ref)_	1.00_(ref)_
2019	1.60 [0.89, 2.87]	1.62 [0.92, 2.84]
2020	2.49 [1.43, 4.33]*	1.97 [1.13, 3.43]*
2021	0.86 [0.43, 1.73]	0.84 [0.43, 1.64]
2022	0.82 [0.42, 1.61]	0.73 [0.38, 1.39]
2023	1.02 [0.57, 1.83]	0.75 [0.44, 1.29]
**Treatment support**:		
No	1.00_(ref)_	1.00_(ref)_
Yes	0.53 [0.37, 0.76]*	0.56 [0.40, 0.79]*
**HIV status**:		
Negative	1.00_(ref)_	1.00_(ref)_
Positive	0.98 [0.57, 1.69]	0.85 [0.53, 1.38]

*Note:* ref = reference category.

* Statistically significant at 95% CI.

## 4 Discussion

The global burden of TB remains a significant public health concern, with disparate geographical incidence patterns and treatment outcomes observed across different regions. This study evidenced the spatial distribution of TB incidence, analysed treatment outcomes over time and identified key predictors of treatment outcomes in Nzema East Municipality, Ghana, providing valuable insights for effective TB interventions and policy formulation and implementation.

### 4.1 Geospatial Distribution of the TB Cases

The significant cluster pattern of TB cases identified through Moran's I autocorrelation in this study suggests that the distribution of TB in Nzema East is non‐random and shows spatial dependence. This variation in TB incidence in Nzema East aligns with previous studies conducted in other regions. For example, the study by Tomita et al. [31] in South Africa also identified significant spatial clustering of TB cases. Additionally, a recent study in Ghana by Iddrisu et al. [32] supports the findings of this current study. Similarly, Abdul et al. [33] found a significant clustering of TB cases in Ghana. This clustering of TB indicates that certain areas within Nzema East are more susceptible to TB transmission. Consequently, the universal approach to TB control is unlikely to be effective. Instead, targeted interventions tailored to the specific characteristics of different geographic areas are crucial in preventing and controlling TB.

The kernel density analysis in this study further corroborated this clustering pattern by identifying areas of highly dense TB incidence across all subdistricts of Nzema East. This ubiquitous incidence of high TB density in at least one settlement in each of the seven sub‐municipalities indicates that TB is a widespread concern throughout the municipality. However, some of the settlements were more burdened with TB than others.

The hotspot analysis yielded noteworthy results, identifying higher hotspot zones. The central hotspot zones were found in Axim and Nsein, urban cluster areas with a high population density. Identifying hotspots in population‐dense areas is consistent with the known transmission dynamics of TB. As indicated by Teibo et al. [34], higher population density is a major predictor of higher TB incidence. Higher population density often correlates with increased close contact between individuals, facilitating the airborne spread of MTB [35]. This finding emphasises the importance of urban planning and housing policies that can mitigate overcrowding and improve ventilation in densely populated areas.

Another significant finding is the presence of hotspots in Bamiankor, Avlebo, Apataim and Fantekrom, small‐scale mining communities in Nzema East Municipality. This aligns with existing research on the heightened TB risk among miners, including those in artisanal and small‐scale gold mining operations [36]. The elevated TB incidence in mining communities can be attributed to several factors, including exposure to silica dust, which increases susceptibility to TB, and higher HIV prevalence in those communities [37]. These findings collectively point to the complex interplay of occupational, environmental and socioeconomic factors in shaping the spatial distribution of TB in the Nzema East.

### 4.2 Trend of TB Treatment Outcomes

Overall, the treatment success rate for the study period was not encouraging, 76.70%, about 13% less than the recommended target by the End TB Strategy [1]. Comparatively, a retrospective study of the 2013 to 2017 cohort conducted in the Volta region of Ghana found an overall TB treatment success rate of 82.5%, which is higher than that recorded in this study but also not up to the recommended 90% minimum success rate by the End TB Strategy [38]. This menace demands immediate strategies for scaling up the treatment outcomes.

The trend of TB treatment outcomes revealed a complex pattern of changes over the study period. A statistically significant decrease in successful TB treatment outcomes was observed from the first half‐year of 2018 to the second half‐year of 2020. This decline coincided with the COVID‐19 pandemic period, which globally disrupted healthcare services [39]. Specifically in Nzema East, this period saw reduced access to TB diagnostic services, treatment monitoring and support services due to COVID‐19‐related restrictions and resource reallocation [14].

The period from late 2020 to early 2021 showed an improvement in treatment success rates, though not statistically significant. This period aligned with the adaptation of TB services to the pandemic context, including the implementation of modified treatment support strategies and enhanced community engagement programs. The non‐significant nature of this improvement suggests that although health systems adapted, the recovery was gradual and faced ongoing challenges from pandemic‐related disruptions.

A significant decrease in successful treatment outcomes was again noted from the first half‐year of 2023 to the second half‐year of 2023. This decline appeared to be driven by different factors than the 2020 decrease, primarily relating to health system capacity constraints in the face of increased case detection (as noted in Section 1.1, there was a 62.34% increase in new cases from 2022 to 2023). This surge in cases likely strained existing treatment support systems and follow‐up mechanisms, impacting treatment adherence and outcomes [40, 41]. As stated in Ghana's 2023 Holistic Assessment Report [42], the Western Region, which includes Nzema East Municipality, had a substantial decline in the human resources relevant for TB treatment, despite the increase in TB case detection. The region's doctor‐to‐population density was 0.09 per 1000 in 2023, down from 0.10 per 1000 in 2021, and nurse‐to‐population density was 1.64 per 1000 in 2023, compared to 1.70 per 1000 in 2021 [42]. Additionally, the municipality's increasing mining activities during this period may have contributed to treatment interruptions due to population mobility and occupational challenges in treatment adherence.

### 4.3 Predictors of TB Treatment Outcomes

Follow‐up laboratory tests during treatment, treatment supporters and the year treatment was initiated emerged as significant factors influencing TB treatment outcomes.

The presence of a treatment supporter was significantly associated with improved treatment outcomes, with patients having treatment supporters showing a 44% lower risk of unsuccessful treatment outcomes (aRR = 0.56; 95% CI = 0.40, 0.79). Treatment supporters in the Nzema East Municipality typically include family members, healthcare workers and community volunteers who assist patients throughout their treatment journey. These supporters play crucial roles in:

1. direct observation of medication intake;
2. providing psychological and emotional support;
3. ensuring attendance at follow‐up appointments;
4. monitoring and reporting adverse reactions.

This finding aligns with a systematic review and meta‐analysis by Alipanah et al. [43], which highlighted the importance of directly observed therapy (DOT) and psychological support in improving treatment outcomes. The significant association between treatment support and successful outcomes underscores the critical role of structured support systems in TB management [44].

Moreover, this study found that patients with at least one follow‐up lab were at 57% less risk of experiencing unsuccessful treatment outcomes, consistent with other research emphasising the importance of regular monitoring (aRR = 0.43; 95% CI = 0.32, 0.58). Regular follow‐up during treatment allows timely identification and management of complications or non‐adherence, thereby improving treatment success rates [45, 46]. The follow‐ups during treatment ensure the patients' contact with healthcare providers, who in turn provide counselling and support.

Although our analysis did not find a statistically significant association between HIV status and TB treatment outcomes (*p* = 0.94), it is important to acknowledge HIV's potential indirect effects on treatment success. HIV affects TB treatment through multiple mechanisms: immune system compromise, increased risk of drug interactions, higher pill burden and potential challenges with treatment adherence due to managing dual conditions. In our cohort, 8.81% of cases were HIV‐positive, which is similar to the global TB–HIV co‐infection rate of 6.1% [1]. Although not statistically significant in our analysis, HIV status remains a clinically important consideration in TB treatment planning and monitoring, particularly in ensuring appropriate support systems and careful management of both conditions. This understanding aligns with broader evidence suggesting that HIV can complicate TB treatment, even when not directly associated with treatment outcomes in statistical analyses [27, 47, 48]. However, our study found that the TB clients co‐morbid with HIV were at 15% less risk of having unsuccessful TB treatment outcome compared to TB clients without HIV infection (aRR = 0.85; 95% CI = 0.53, 1.38). This could be the result of the implementation of the TB/HIV integrated care in Nzema East, Ghana. Ghana's implementation of TB/HIV collaborative activities since 2007 has led to significant improvements in the management of TB/HIV co‐infection. This integrated approach, aligned with WHO recommendations, has potentially reduced the risk of unsuccessful TB treatment outcomes among HIV‐positive individuals [49, 50, 51].

## 5 Conclusion and Recommendations

### 5.1 Conclusion

The study has revealed a concerning spatial clustering of TB cases within Nzema East Municipality, characterised by highly dense TB incidence. Notably, hotspot zones were identified in densely populated and mining communities, highlighting the need for targeted interventions. The study further propounded a concerning decline in successful TB treatment outcomes, which fell below the recommended targets of the End TB Strategy. This decline was evident from the first half of 2018 to the end of 2020 and from the first to the second half of 2023. Factors such as follow‐up laboratory tests, treatment support and the year treatment commenced were statistically significant predictors of TB treatment outcomes. These findings highlight the complex interplay of socio‐demographic, environmental and programmatic factors influencing TB transmission and treatment success, underscoring the importance of addressing the spatial and temporal factors in TB control efforts.

### 5.2 Limitations of the Study

1. The complete use of secondary data for this study may have introduced potential biases due to the data accuracy. However, the data were extracted twice by two groups, which were validated, and any errors found were duly amended as appropriate. This would limit the potential biases that could have been introduced during the data collection.
2. The study may not have fully accounted for potential confounding variables due to the sole use of secondary data, which does not capture the wider determinants.
3. The relatively small sample size of 545 TB cases over a 6‐year period may limit the reliability of the observed year‐by‐year changes in treatment outcomes. With an average of fewer than 100 cases per year, the annual trends should be interpreted with caution, as minor fluctuations in case numbers could substantially impact the proportions of treatment outcomes.

### 5.3 Recommendations

#### 5.3.1 For Policymakers

1. Implement focused TB control measures in hotspot zones, especially in densely populated and mining communities, including increased screening, awareness campaigns and community engagement to reduce transmission rates.
2. Strengthen the role of TB treatment supporters to improve adherence and treatment success. Training and deploying community health workers to provide support and follow‐up can be crucial in these high‐risk areas.
3. Ensure that patients have at least one follow‐up laboratory test during their treatment. This can help detect adverse treatment outcomes early and adjust therapy as needed.
4. Incorporating multisectoral approaches, such as involving mining companies in formulating and implementing TB control strategies.
5. Integrate real‐time GIS‐based dashboards for TB surveillance and management in the health facilities into existing health information systems such as the DHIS2 and SORMAS for timely detection of TB hotspots and data‐driven decision‐making.

#### 5.3.2 For Future Research

1. Prospective studies using primary data are recommended in future research to identify the wider determinants of TB treatment outcomes.
2. Future research should expand the geographical area and use a longer period to capture more comprehensive trends and variations in TB incidence and treatment outcomes.

## Author Contributions

**Charles Afriyie Agyapong**: conceptualisation, investigation, methodology, writing – review and editing, formal analysis, software, data curation, writing – original draft. **Ali Davod Parsa**: methodology, writing – review and editing, project administration, supervision, resources, writing – original draft. **Richard Hayhoe**: visualisation, formal analysis, writing – review and editing, methodology, software, supervision, resources, project administration, writing – original draft. **Russell Kabir**: methodology, validation, visualisation, writing – review and editing, software, formal analysis, data curation, supervision, resources, writing – original draft. **Mark Cortnage**: methodology, writing – review and editing, writing – original draft, formal analysis, project administration, supervision.

## Conflicts of Interest

The authors declare no conflicts of interest.

## Data Availability

The data that support the findings of this study are available from the corresponding author upon reasonable request.
